# Longitudinal analysis of peripheral blood T cell receptor diversity in patients with systemic lupus erythematosus by next-generation sequencing

**DOI:** 10.1186/s13075-015-0655-9

**Published:** 2015-05-23

**Authors:** Dharma R Thapa, Raffi Tonikian, Chao Sun, Mei Liu, Andrea Dearth, Michelle Petri, Francois Pepin, Ryan O Emerson, Ann Ranger

**Affiliations:** Biogen, 250 Binney Street, Cambridge, MA 02142 USA; Novartis Pharmaceuticals Canada Inc, 385 Bouchard Boulevard, Dorval, QC H9S 1A9 Canada; Johns Hopkins University School of Medicine, 1830 East Monument Street, Baltimore, MD 21205 USA; Adaptive Biotechnologies, 1551 Eastlake Avenue East, Seattle, WA 98102 USA

## Abstract

**Introduction:**

T cells play an important role in the pathogenesis of systemic lupus erythematosus (SLE). Clonal expansion of T cells correlating with disease activity has been observed in peripheral blood (PB) of SLE subjects. Recently, next-generation sequencing (NGS) of the T cell receptor (TCR) β loci has emerged as a sensitive way to measure the T cell repertoire. In this study, we utilized NGS to assess whether changes in T cell repertoire diversity in PB of SLE patients correlate with or predict changes in disease activity.

**Methods:**

Total RNA was isolated from the PB of 11 SLE patients. Each subject had three samples, collected at periods of clinical quiescence and at a flare. Twelve age-matched healthy controls (HC) were used for reference. NGS was used to profile the complementarity-determining region 3 (CDR3) of the rearranged TCR β loci.

**Results:**

Relative to the HC, SLE patients (at quiescence) demonstrated a 2.2-fold reduction in repertoire diversity in a given PB volume (*P* <0.0002), a more uneven distribution of the repertoire (Gini coefficient, HC vs SLE, *P* = 0.015), and a trend toward increased percentage of expanded clones in the repertoire (clone size >1.0 %, HC vs SLE, *P* = 0.078). No significant correlation between the overall repertoire diversity and clinical disease activity was observed for most SLE patients with only two of eleven SLE patients showing a decreasing trend in repertoire diversity approaching the flare time point. We did not observe any overlap of CDR3 amino acid sequences or a preferential Vβ or Jβ gene usage among the top 100 expanded clones from all SLE patients. In both HC and SLE, the majority of the expanded clones were remarkably stable over time (HC = 5.5 ±0.5 months, SLE = 7.2 ±2.4 months).

**Conclusions:**

A significant decrease in T cell repertoire diversity was observed in PB of SLE patients compared to HC. However, in most SLE patients, repertoire diversity did not change significantly with increases in disease activity to a flare. Thus, without *a priori* knowledge of disease-specific clones, monitoring TCR repertoire in PB from SLE patients is not likely to be useful to predict changes in disease activity.

**Electronic supplementary material:**

The online version of this article (doi:10.1186/s13075-015-0655-9) contains supplementary material, which is available to authorized users.

## Introduction

Systemic lupus erythematosus (SLE) is a prototypic autoimmune disorder with complex etiology, diversity of clinical manifestations, and an unpredictable disease course. T cells play an essential role in SLE pathogenesis [1–5]. Clonal expansion of T cells have been observed in SLE patients’ peripheral blood (PB) [6–12] and various organs such as skin [13], kidney [8, 14–16] and gastrointestinal tract [17] where they may be reactive to local antigens and drive tissue inflammation and injury. *In vitro* studies show that T cells from SLE patients can recognize and proliferate in response to specific autoantigens such as nucleosomal histones [2, 18] and U1 small nuclear ribonucleoprotein A [19, 20]. Furthermore, murine SLE models show T cell expansions [21–24], the dependence of pathogenic anti-DNA autoantibodies production on CD4+ T cells [25–27], and slowed disease progression as a result of T cell depletion [28]. Taken together, these studies suggest a crucial role for T cells in the pathogenesis of SLE.

Ninety-five percent of T cells in the blood express T cell receptor (TCR) consisting of heterodimers of αβ chains [29]. TCRβ chains are assembled by combinatorial somatic recombination events that splice together the variable (V), diversity (D), and joining (J) gene segments (VDJ) [29]. This junctional region of the TCR chains, also known as the complementarity-determining region 3 (CDR3), is highly diverse and is an important determinant of antigen recognition by T cells. Because allelic exclusion leads to the expression of only one TCR chain in mature T cells [29], each unique CDR3 sequence is a proxy for T cell clonotype. Thus, analysis of TCR CDR3 sequences has provided a useful means to study clonal expansions, repertoire breadth, and other properties such as CDR3 length polymorphisms, V(D)J gene usage, and sequence specificity of the T cell response.

Prior studies examining the expansion and diversity of the TCR repertoire in SLE have used reverse transcriptase-polymerase chain reaction (RT-PCR) of the TCRβ CDR3 region followed by techniques such as Southern blots [13, 14], CDR3 spectratyping [11] and immunoscope analysis [6, 9], single-strand conformation polymorphism (SSCP) [10, 13], or laborious cloning and sequencing analysis of select bands (or peaks) [6–8, 12, 14, 15]. Several interesting observations have come out of these studies, such as clonal expansions and reduction of the TCRβ repertoire diversity in PB [6, 7, 9, 11], correlation of PB T cell expansions [8, 10] or spectratype skewing [6] with disease activity, and the abundance of clonally expanded T cell populations in skin lesions [13] and kidneys [14–16] with concomitant detection of overlapping clones in PB [15] or not [13, 14, 16]. The immensity of T cell diversity presents a formidable challenge to its study and the aforementioned techniques lack sufficient depth of coverage (ability to detect low-frequency clones) and resolution (lack of sequence information without additional cloning steps). The advent of next-generation sequencing (NGS) has provided a means to exhaustively sequence and accurately quantify the relative abundance of TCR sequences within a given sample in a high-throughput manner. Consequently, NGS has become a powerful tool in the study of immune repertoire with potential applications in the identification and tracking of pathogenic clones, monitoring immune status and disease activity, and immune reconstitution following therapeutic interventions.

Disease flares in SLE patients are a challenge to clinical management as they are difficult to predict. The objective of this study was to use NGS to perform a longitudinal analysis of the T cell repertoire in blood from SLE patients prior to and at an increase in clinical disease activity. The CDR3 region of the TCRβ chain was sequenced from the blood of 11 SLE patients over three visits each, representing clinical quiescence, pre-flare, and flare stages. We defined flare as an increase in SLE disease activity index (SLEDAI) ≥4 and/or an increase in the Physician Global Assessment (PGA) of ≥1. Twelve healthy controls (HC), including six subjects with PB collected at two longitudinal time points, were included for comparison. Our findings show a significant reduction in T cell repertoire diversity in SLE patients compared to HC. However, in most cases, an increase in SLE disease activity leading to a flare was not accompanied by significant changes in the TCR repertoire diversity.

## Methods

### Patient selection and controls

All SLE subjects were selected from an observational study, the Study of biological Pathways, disease Activity and Response markers in patients with systemic lupus Erythematosus (SPARE). The study was approved by the Johns Hopkins University School of Medicine Institutional Review Board. SLE patients were enrolled from the Hopkins Lupus Cohort following informed consent. Adult patients were eligible if they were aged 18 to 75 years old and met the definition of SLE as defined by the revised American College of Rheumatology classification criteria [30, 31]. All patients were evaluated by the same physician at entry and all subsequent cohort visits (MP). Three longitudinal samples were selected from 11 female SLE patients. One sample was chosen at clinical flare, defined as an increase in Safety of Estrogens in Lupus Erythematosus National Assessment (SELENA) SLEDAI from the preceding visit by ≥4 (maximum increase = 18) and/or an increase in PGA of ≥1.0 (maximum increase = 2.0). For each SLE subject, two samples were chosen at visits with no clinical activity as defined by SELENA SLEDAI <2 (low complement or double-stranded DNA (dsDNA) only) and/or PGA ≤0.5. The visit immediately preceding the flare was defined as pre-flare. The average duration from quiescence to flare was 7.2 ±2.4 months (± standard deviation (SD), range of 4.6 to 13.8 months) whereas the average duration between any consecutive sample points was 3.6 ±1.9 months. The severity of the flare for the 11 SLE patients was varied. Using the following SLEDAI-based stratification (0 = no increase, 1 to 5 = mild, 6 to 10 = moderate, 11 to 19 = high, and ≥20 = very high) [32], only one of eleven flared at a high level, seven of eleven at moderate level, and two of eleven at mild level. One patient (SLE7) exhibited no increase in SLEDAI score at flare, but was selected based on an increase in PGA from 0 to 1.5. Organ involvement during flare was selected to include renal (n = 4), joint (n = 3), and skin (n = 4). All patients were taking immunosuppressant(s) and/or antimalarial drugs (hydroxychloroquine) at the time samples were obtained. The demographic and clinical characteristics of these patients are summarized in Table 1. Twelve age- and race-matched healthy female volunteers were included as HC (from Bioreclamation LLC, Hicksville, NY, USA and the Biogen blood donor program in accordance to IRB protocol 20124572). For six of the HC subjects, two longitudinal samples (average duration = 5.5 ±0.5 months) were analyzed.

**Table 1:** Systemic lupus erythematosus (SLE) subject characteristics

Subject/ethnicity	Disease stage/organ involved	Elapsed time (months)	PGA	SLEDAI	WBC (10^3^/mm^3^)	C3 (mg/dl)	C4 (mg/dl)	Anti-dsDNA (U/ml)	Therapy
SLE1/white	Quiescent	0	0	2	3.93	146	22	40	MMF/HCQ
	Pre-flare	4	0	0	3.82	108	19	0	MMF/HCQ
	Flare (skin)	6	1.5	10	4.34	115	21	80	MMF/HCQ
SLE2/white	Quiescent	0	0	2	5.6	85	2	0	Pred/MMF
	Pre-flare	3	0.5	2	5.5	88	3	0	Pred/MMF
	Flare (skin)	5	2	10	7.8	105	4	0	Pred/MMF
SLE3/white	Quiescent	0	0.5	2	6.26	86	11	0	Pred
	Pre-flare	5	0.5	2	7.04	86	11	0	Pred
	Flare (skin)	7	2	10	4.53	85	11	0	Pred
SLE4/white	Quiescent	0	0	0	6.44	96	21	0	Pred/AZA
	Pre-flare	3	0	0	8.13	106	22	0	Pred/AZA
	Flare (joint)	6	1.5	4	7.96	102	22	0	Pred/AZA
SLE5/white	Quiescent	0	0	0	2.68	108	21	0	MMF/HCQ
	Pre-flare	3	0	0	1.78	98	17	0	MMF/HCQ
	Flare (renal)	7	1.5	10	1.85	81	13	40	MMF/HCQ
SLE6/black	Quiescent	0	0	0	13.35	134	19	0	Pred/HCQ
	Pre-flare	3	0.5	0	9.16	135	26	0	HCQ
	Flare (renal)	6	1.5	4	10.35	121	21	0	HCQ
SLE7/black	Quiescent	0	0	0	5.3	134	35	0	AZA/HCQ
	Pre-flare	3	0	0	6.3	139	38	0	AZA/HCQ
	Flare (joint)	14	1.5	0	4.4	145	40	0	AZA/HCQ
SLE8/white	Quiescent	0	0	0	5.53	106	21	0	HCQ
	Pre-flare	4	0	0	5.83	97	17	0	HCQ
	Flare (skin)	7	1	18	4.72	94	18	0	HCQ
SLE9/black	Quiescent	0	0	0	5.75	161	39	0	Pred/MMF
	Pre-flare	3	0	0	5.83	145	29	0	Pred/MMF
	Flare (renal)	9	1.6	6	7.08	186	29	0	Pred/MMF
SLE10/black	Quiescent	0	0.5	2	7.3	144	60	0	Pred/HCQ
	Pre-flare	4	0.5	0	6.93	90	38	0	Pred/HCQ
	Flare (renal)	6	2.5	6	6.62	97	43	0	Pred/HCQ
SLE11/white	Quiescent	0	0.5	0	7.26	146	27	0	Pred/LFM
	Pre-flare	3	0	0	7.14	132	33	0	Pred/LFM
	Flare (joint)	7	1.5	6	7.18	139	26	0	Pred/LFM

All subjects were female. Reference ranges, WBC: 4.5 to 11 x10^3^/uL; C3: 79 to 152 mg/dL; C4: 14 to 42 mg/dL; dsDNA titer <10 considered negative. AZA, azathioprine; C3, complement component 3; C4, complement component 4, dsDNA, double-stranded DNA; HCQ, hydroxychloroquine; LFM, leflunomide; MMF, mycophenolate mofetil; PGA, Physician Global Assessment; Pred, prednisone; SLEDAI, SLE disease activity index; WBC, white blood cell

### RNA isolation and cDNA synthesis

Total RNA was isolated from PAXgene preserved PB using the PAXgene blood RNA kit (PreAnalytiX, Franklin Lakes, NJ, USA). cDNA was prepared from 500 ng of total RNA using the high-capacity cDNA reverse transcription kit (Applied Biosystems, Foster City, CA, USA).

### TCRβ repertoire sequencing and analysis

NGS was performed using the immunoSEQ platform (Adaptive Biotechnologies, Seattle, WA, USA), and the methodology used has been validated and detailed before [33]. Briefly, the CDR3 region of the TCRβ loci was amplified from cDNA using PCR primers specific for all Vβ and Jβ genes annotated in the IMGT database [34], barcoded, and deep-sequenced using the Illumina HiSeq platform (Illumina, San Diego, CA, USA). Repertoire diversity was assessed using clonality scores, which is derived from Shannon’s entropy [35]. Shannon’s entropy quantifies the uncertainty in predicting the sequence identity of a random sequence from a dataset. To allow for comparisons between samples differing in the total number of reads, entropy was normalized by division of log 2 of the number of unique productive sequences. Clonality is the reciprocal of normalized Shannon’s entropy with values ranging from 0 (most diverse) to 1 (least diverse). Gini coefficient, commonly used as a measure of income inequality in economics, was used to assess the inequality of clonotype distribution within a repertoire [36]. The Lorenz curve was derived by plotting the cumulative proportion of total sequences on the y-axis relative to contribution by unique clonotypes on the x-axis. Gini coefficient, defined as the ratio of the area between the line of equality (45 degree line) and the Lorenz curve to the area under the line of equality, was calculated using the trapezoidal area method [37]. Gini coefficient value ranges from 0 representing equal frequency of all clonotypes in the repertoire to 1 representing a monoclonal sample.

### Statistical analysis

Statistical analysis was performed using GraphPad Prism v.6 (GraphPad Software, San Diego, CA, USA). Nonparametric comparisons between groups were performed using Mann–Whitney test (two groups), and between paired samples using Wilcoxon (two groups) or Friedman test (three groups). Mean values were compared using *t* test. Correction for multiple testing was performed using false discovery rate (FDR) set to 10 %. *P* <0.05 was considered significant. Motif analysis was performed using HOMER DNA motif analysis software [38], using the top 100 CDR3 sequences from SLE patient samples as query and the top 100 CDR3 sequences from HC as background. Significantly expanded or contracted T cell clones across longitudinal samples were defined by using DEseq R package to train a model of T cell repertoire dispersion over time using the HC samples and then applied to all samples with a *P* value cutoff of 0.001, as previously described [39, 40].

## Results

### Patient summary

The demographic and clinical characteristics of the 11 female SLE patients are summarized in Table 1. Patient selection criteria are described in Methods. For comparative analysis, 12 female HC were included in this study. There was no significant difference in age between the two groups (years ±SD of 42.2 ±9.0 (HC) vs 46.1 ±7.9 (SLE), *P* = 0.291). Racial composition was 67 % white and 33 % black in HC vs 63 % white and 37 % black in SLE. All SLE subjects were receiving treatment.

### TCR repertoire diversity and clonal expansions in HC and SLE subjects

**Fig. 1:**
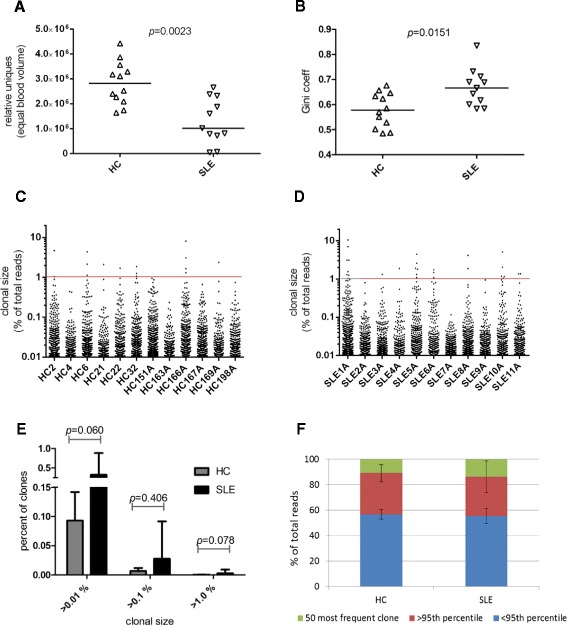
Analysis of T cell receptor (TCR) repertoire of healthy controls (HC) and systemic lupus erythematosus (SLE) patients. **a** Repertoire diversity as measured by the number of clonotypes per normalized blood volume, HC vs SLE (Mann–Whitney test). **b** Evenness of clonotype distribution using Gini coefficient, HC vs SLE (Mann–Whitney test). **c** and **d** Scatterplot of clonotypes exceeding >0.01 % clonal size of the total reads, for HC (C) and SLE (D). Clonal size >1 % (red horizontal line) designates highly expanded clones (HEC). **e** Frequency distribution of clones by size difference between HC and SLE. Mean values with error bars representing + standard deviation (SD) are plotted for clonal sizes >0.01 %, >0.1 %, and >1.0 % (Mann–Whitney test). **f** Proportion of total repertoire occupied by clonal groups defined by the top 50, the >95^th^ percentile and the <95^th^ percentile of clones. Mean values with error bars representing ±SD are shown. HC vs SLE; top 50 clones group, *P* = 0.913, >95^th^ percentile group, *P* = 0.913 (Mann–Whitney test)

Deep level sequencing of the TCRβ CDR3 region from PB of 23 subjects (51 samples) produced an average of 6,472,081 total and 317,535 unique productive reads (Table S1 in Additional file 1). First, we wanted to assess any groupwise differences in repertoire diversity between HC and SLE. Only patient samples corresponding to disease quiescence were utilized for this comparison with HC. Because each unique CDR3 sequence denotes a T cell clonotype, we assessed quantitative difference in the number of uniques between HC and SLE. We found that the number of uniques when normalized to a fixed blood volume was 2.2-fold lower in treatment-experienced SLE subjects compared to HC subjects (*P* = 0.0023, Fig. 1a). Using Gini coefficient, we also found a significant trend toward a more skewed clonotype distribution (higher values) in SLE compared to HC (*P* = 0.0151, Fig. 1b). Thus, for a given volume of PB, SLE repertoire is significantly diminished in diversity and skewed in its distribution.

Next, we wanted to assess the relative degree of clonal expansions within the repertoire of these two groups. The size of each clone was calculated based on its frequency in the repertoire. Scatterplots of all clones >0.01 % are shown in Fig. 1c (HC) and Fig. 1d (SLE). There was no significant difference in the percentage of clones in the repertoire that were >0.01 % (*P* = 0.060), >0.1 % (*P* = 0.406), or >1.0 % (*P* = 0.078) between HC and SLE, although SLE subjects consistently showed a higher percentage of these clonally expanded populations (Fig. 1e). When the cumulative contribution by the top 500 clones to the total repertoire was analyzed, we found disproportionately larger contribution by just the top 50 clones (Figure S1 in Additional file 2). We therefore placed the clones into three groups based on their size - the top 50 clones, >95^th^ percentile, and <95^th^ percentile for comparative analysis (Fig. 1f). There was no significant difference in the percent contribution to the repertoire by the top 50 clones between HC and SLE subjects (average percent of repertoire ±SD of 10.85 ±6.75 (HC) vs 13.62 ±12.37 (SLE), *P* = 0.913) or by the >95^th^ percentile group (average percent of repertoire ±SD of 43.21 ±6.81 (HC) vs 44.77 ±11.29 (SLE), *P* = 0.913). Thus, in both HC and SLE groups, the top 5^th^ percentile of clones is highly expanded and accounts for >40 % of the repertoire.

Preferential use of TCR Vβ genes in SLE subjects has been reported in PB [6] or intrarenal T cells [14], while other studies have shown no such preference in PB [8, 10]. We calculated the frequency of Vβ and Jβ gene usage as a percentage of uniques to avoid bias from expanded clones. We found differential usage of four Vβ genes: V05-04, V05-05, V05-06, V21-01 (HC vs SLE, all comparisons *P* <0.05, FDR = 10 %) and four Jβ genes: J01-02, J01-04, J01-05, J02-01 (HC vs SLE, all comparison *P* <0.05, FDR = 10 %) (Figure S2 in Additional file 2). To account for potential contribution of race to these results, groupwise comparison of gene usage was performed between whites and blacks. None of the genes were differentially expressed by race.

### Longitudinal evaluation of the TCR repertoire in HC and SLE subjects

**Fig. 2:**
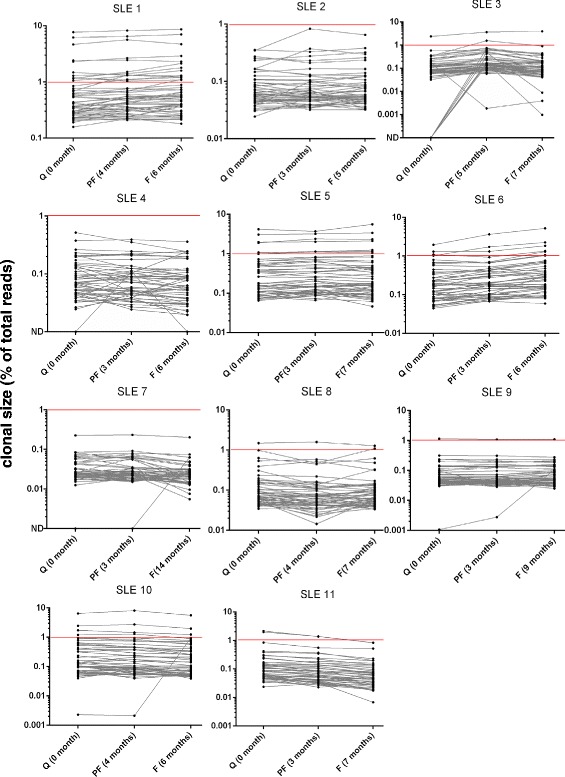
Longitudinal analysis of T cell receptor (TCR) repertoire in systemic lupus erythematosus (SLE) patients. Clonotype frequency and relative ranks of the top 50 clones in SLE. The three time points in SLE patients represent quiescence (Q), pre-flare (PF) and flare (F). The elapsed time (months) from the first sample are indicated. Clones above the horizontal red line (>1 %) denotes highly expanded clones (HEC)

**Fig. 3:**
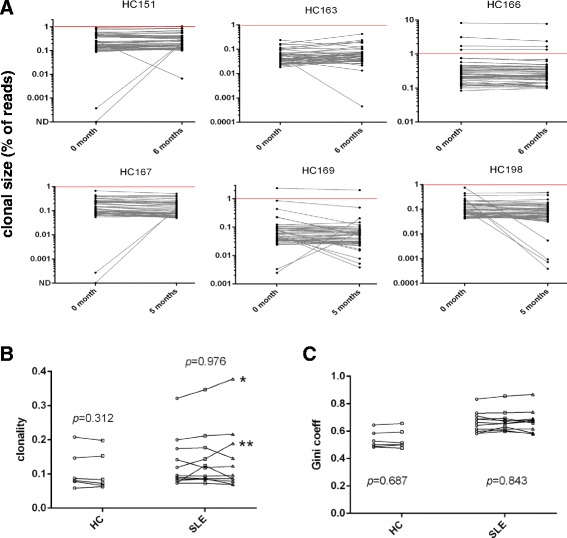
Longitudinal analysis of T cell receptor (TCR) repertoire in healthy control (HC) subjects and comparisons with systemic lupus erythematosus (SLE) patients. **a** Clonotype frequency and relative ranks of the top 50 clones in HC. The elapsed time (months) from the first sample are indicated. Clones above the horizontal red line (>1 %) denotes highly expanded clones (HEC). **b** and **c** Analysis of clonality (b) and Gini coefficient (c) values in longitudinal samples from HC and SLE patients. No significant trends for clonality or Gini coefficient values were observed within either HC or SLE samples (HC, paired analysis using Wilcoxon test; SLE, paired test using Friedman test). Only patients SLE1^*^ and SLE6^**^ exhibited an increase in clonality (decreasing diversity) at clinical flare. Longitudinal data points from each individual are connected by a line

To investigate the differences in repertoire diversity during increase in clinical disease activity, we looked at longitudinal samples representing quiescence, pre-flare, or flare status for all 11 SLE patients over an average duration of 7.2 months (range, 4.6 to 13.8 months). For reference, six HC repertoires were analyzed over an average period of 5.5 months (range, 5 to 6 months). Changes in the frequency and rank of the top 50 clones between the time points for each subject are shown in Fig. 2 (for SLE) and Fig. 3a (for HC). The top 50 clones were highly stable with 73 % (eight of eleven) of SLE and 67 % (four of six) of HC subjects showing no addition or subtraction of clones over time. A single clonotype addition into the top 50 repertoire was seen in 18 % (two of eleven) of SLE and 33 % (two of six) of HC. Only one of eleven (9 %) SLE subjects (SLE3) showed appearance of seven new clones during the pre-flare time point (Fig. 2). The amino acid sequence, rank, and frequency of the top 50 clones in the repertoire are summarized in Table S2 in Additional file 1 for HC and Table S3 for SLE.

Several other observations stand out with regard to clonal expansions in PB. We find that most repertoires are dominated by only a few clones whose abundance and rank is maintained over time. Using clone size >1 % as a reference for highly expanded clones (HEC), a term coined by Klarenbeek *et al*. [41], 73 % (eight of eleven) of SLE subjects contained at least one clone of size >1 % in their repertoire (Fig. 2). However, HECs can also be seen in HC where 33 % (two of six) subjects (HC166 and HC169) showed clones >1 % in their repertoire (Fig. 3a). Thus HEC is not a feature unique to SLE and can also be observed in HC. Next, using clonality as a measure of repertoire diversity, we assessed how the overall T cell diversity in patients changes during increase in disease activity to a flare. Clonality scores showed a wide distribution of values between subjects within both groups (Fig. 3b). However, within each subject, no significant changes in clonality were seen in longitudinal samples from either HC (*P* = 0.312) or SLE groups (*P* = 0.976). Only two of eleven SLE subjects (SLE1 and SLE6, marked as ^*^ and ^**^ in Fig. 3b) showed increasing clonality (corresponding to decreasing diversity) going from disease quiescence to flare. Next, we used Gini coefficient to look at changes in the evenness of clonal distribution of the repertoire over time. No significant changes in Gini coefficient were seen between longitudinal samples of HC (*P* = 0.687) or SLE (*P* = 0.843) subjects (Fig. 3c), although Gini coefficient differed significantly between the two groups (Fig. 1b). In order to assess whether increased clonal expansion occurred during flare, we also looked at changes in the relative contribution to the total repertoire by the three groups of clones stratified by size (the top 50, >95^th^ percentile, and <95^th^ percentile). No significant difference in contribution to the total repertoire by these three groups of clones was seen in longitudinal samples from SLE (Figure S3 in Additional file 2) or HC subjects (Figure S4 in Additional file 2). Again, when analyzed individually, the exceptions were two SLE subjects (SLE1 and SLE6) who showed a steady increase in the percentage of repertoire contributed by the top 50 and the >95^th^ percentile group (Figure S3 D and E in Additional file 2) mirroring the clonality results (Fig. 3b).

Finally, we investigated the expansion and contraction of T cell clonal lineages between quiescent and flare samples in SLE patients and compared these to the six pairs of HC samples. Clones were identified as expanded or contracted if their abundance in the flare sample was different from their abundance in the quiescent sample at *P* <0.001 using a DEseq differential abundance model trained on the six HC pairs (see Methods). We found a median of 15 (range 0 to 165) expanded clones and 9 (range 4 to 23) contracted clones in the HC pairs, and a median of 9 (range 0 to 114) expanded clones and 2 (range 0 to 19) contracted clones in the SLE sample pairs; thus, the clonal dynamics revealed by a differential abundance analysis do not support an excess in the clonal expansion of T cell lineages in SLE patients from quiescence to flare compared to HC sampled at similar time points. Together, these data suggest that in SLE subjects, increase in clinical disease activity is not always accompanied by overt expansion of T cell clones and concomitant decrease in repertoire diversity in the blood.

### CDR3 sequence diversity of dominant clones from SLE patients

**Fig. 4:**
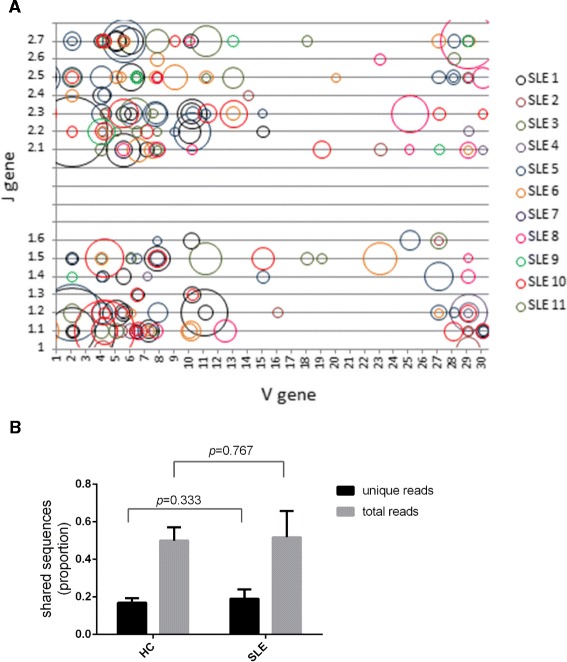
Analysis of primary structure of T cell receptor (TCR**)** β complementarity-determining region 3 (CDR3) sequences. **a** Vβ and Jβ gene usage by expanded clones (size >0.1 %) from systemic lupus erythematosus (SLE) patients. The area of each bubble corresponds to the relative size of each clone. None of the CDR3 amino acids sequences were overlapping. **b** The amount of sequences that overlap in the repertoire across time points as a proportion of unique reads or total reads in healthy controls (HC) and SLE patients. Mean values with error bars depicting + standard deviation (SD) are shown (Mann–Whitney test)

Next, we wanted to address whether clonally expanded T cells in SLE shared any common structural features between patients. We found great heterogeneity and minimal overlap in the use of Vβ and Jβ gene by the top clones (>0.1 % by size) from the 11 SLE repertoires (Fig. 4a). Where the Vβ and Jβ gene usage overlapped, the amino acid sequence of the CDR3 region was unique. In fact, we did not find any overlap in the amino acid sequences of the top 100 clones (which is a more encompassing list than that generated by >0.1 % size criteria) between any SLE patients. Similarly, no overlap was seen for the top 100 clones in HC subjects. This is in agreement with prior studies in SLE blood showing no CDR3 amino acid sequence overlap of the expanded T cell clones between subjects [6, 8]. Next, using the HOMER motif-finding module, no amino acid motifs were found to be significantly enriched in the top 100 productive sequences from SLE when compared to HC as the background dataset.

Finally, we addressed the idea that the SLE repertoire may be under selective pressure to maintain autoantigen-reactive clones and consequently, might display a higher proportion of clones that are maintained over time when compared to HC. We found in SLE patients that 19.1 ±4.9 % of clones were shared over time accounting for 51.7 ±13.9 % of the total repertoire, which was not significantly different from HC where 16.8 ±2.4 % of clones were shared over time and accounted for 49.9 ±7.1 % of the total repertoire (HC vs SLE, *P* = 0.333 for unique reads and *P* = 0.767 for total reads, Fig. 4b). In this comparison, the average time between the two sample points for SLE was 4.02 ±2.69 months and for HC was 5.47 ±0.17 months. Thus, compared to HC, SLE repertoire did not display a significantly higher level of clones that were maintained in the repertoire over a similar time period.

## Discussion

One of the key unmet needs in SLE clinical management are biomarkers predictive of flare. It has been well established that T cells play a critical role in the underlying pathogenesis of SLE. Thus, in this study we asked whether changes in the T cell repertoire in the blood of SLE patients as measured by NGS could yield important information about the underlying disease process and potentially serve as a biomarker of disease activity. Published work assessing T cell repertoire in SLE patients has mainly relied on approaches such as Southern blots [13, 14], SSCP analysis [10, 13], and conventional spectratyping [6, 9, 11], which can broadly inform on the V (or J) gene-level usage but requires additional PCR cloning and sequencing steps [6–8, 14, 15] to achieve clonotype-level resolution. Comprehensive assessment of the T cell repertoire entails knowing both the diversity (the number of unique clonotypes) and the relative abundance of these clonotypes (number of reads per clonotype), which can be obtained simultaneously by NGS using genomic DNA (gDNA) or mRNA templates. Although differences in TCRβ mRNA quantity per cell exist [42, 43], clonal rank derived from mRNA abundance correlates well with those quantified from sorted cell population [42, 44] or its corresponding gDNA [45]. Therefore, in this study we used mRNA extracted from blood to evaluate the T cell repertoire of HC and SLE subjects.

Previous studies have shown a higher number of expanded T cell clones in the PB of SLE subjects during active disease [6–8, 10, 11]. While we also found expanded clones in SLE patients, their number and relative size, in most cases, did not significantly increase prior to or during a clinical flare. Several differences in study design, the benefits of NGS notwithstanding, should be noted. The degree of disease activity for most patients in our cohort was moderate (SLEDAI = 6 to 10) with only one patient (SLE8) exhibiting high (SLEDAI = 11 to 19) activity during flare and only two patients (SLE1, SLE5) with a measurable anti-dsDNA antibody titer in serum (Table 1). The study by Kato *et al.* [8] used cloning and sequencing of only three TCRβ families (5S1, 8, 14) to track clonal expansion and found an increase in the proportion of expanded clones in one subject from 24 % to 79 % of total sequences (pre-flare to flare, SLEDAI increase from 4 to 11, anti-dsDNA antibody titer at flare approximately 90 IU/ml). No differences in clonal expansions were seen between times pre- and post-flare. In another study, three patients treated only with prednisolone (Pred) showed a correlation between the number of expanded T cell clones and SLEDAI (with SLEDAI values ranging from 15 to 21 during disease activity and between 2 and 5 during disease inactivity in these subjects) [10]. In this study, the number of distinct bands from SSCP blots was used to indicate clones, however, the homogeneity of such bands or spectratype peaks [6] cannot be assumed without further sequencing. In other studies, cross-sectional comparisons of patients with inactive vs active disease were used to arrive at similar conclusions [7, 9, 11]. Using improved sensitivity of NGS, we find that in most SLE subjects (nine of eleven), flaring to moderate disease activity level was not associated with an increase in clonal expansion or a decrease in overall repertoire diversity. All SLE subjects, with one exception (SLE8), were being treated with immunosuppressants and this could affect the clonal expansion of T cells. However, for each patient, medication was constant between periods of pre-flare and clinical flare and if T cell clonal expansion was key to the increased clinical activity, it should be observed despite treatment. The two patients where increased clinical activity was associated with clonal expansions presented with diverse backgrounds. SLE1 was a white female presenting with a cutaneous flare, on hydroxychloroquine (HCQ) and mycophenolate mofetil (MMF) treatment, and with anti-dsDNA antibodies (80U/ml); SLE6 was a black female presenting with a renal flare, on HCQ treatment, and with undetectable anti-dsDNA antibodies. Because we wanted to capture the diversity of SLE clinical manifestations, our study is not adequately powered to allow analysis of patient subgroups based on flare type, laboratory parameters, or treatments.

By itself, the presence of stable, highly expanded T cell populations in PB is not a feature indicative of a disease state. Rather, even among healthy individuals, T cell responses to vaccination [46] and pathogens [46, 47], particularly against latent viruses such as human cytomegalovirus and Epstein-Barr virus, have been known to occupy a significant proportion of the repertoire for decades [44]. In our study, the top 50 clones represented 10.8 ±6.7 % of the repertoire in HC (vs 13.6 ±12.3 % in SLE, *P* = 0.913) whereas the top 5^th^ percentile of the clones represented 43.2 ±6.8 % of the repertoire in HC (vs 44.7 ±11.3 % in SLE, *P* = 0.913) (Fig. 1f). HC repertoires with an abundance of HECs (clone size ≥1 %) were observed in three of six HC subjects (HC151, HC166 and HC169) among whom HC151 and HC166 had the two highest clonality scores and Gini coefficient values that were comparable to most SLE subjects (Fig. 3a, b, c). Additionally, even though the average proportion of clonally expanded T cells differed among individuals (Figure S3, S4 in Additional file 2), the composition and relative rank of these clones stayed remarkably stable over time (Figs. 2, 3a). Given these data, it is likely that only disease-specific autoreactive clones within the repertoire will display expansion kinetics that correlate with disease activity. Thus, the utility of NGS as a tool to monitor disease activity in SLE could significantly benefit from prior knowledge of such pathogenic clones to enable their tracking. Further studies are warranted using sorted T cell subsets both from blood and affected tissues simultaneously [14, 15, 41] to help pinpoint such disease-specific clones.

Multiple individuals sharing a common TCR, termed public T cell response, has been observed during viral infections, tumorigenesis, and in autoimmune diseases such as multiple sclerosis, rheumatoid arthritis, aplastic anemia, and sarcoidosis (reviewed in [48]). In SLE, the amino acid sequences of the expanded TCR response between individuals are largely non-overlapping. However some studies have shown recurrent T cell CDR3 motifs such as GGX in PB [6], SSG, GQG, and VRG in kidneys [14], LXG in skin [13] or charged residues in PB-derived anti-dsDNA inducing T helper cells [5]. In our study, no overlap in CDR3 sequences was found between any SLE patients when the top 100 clones from each repertoire were compared. While this suggests a general lack of public response in PB of SLE patients, several considerations should be made with this interpretation. First, TCR repertoire is shaped by the human leukocyte antigen (HLA) type of the individual. However, due to sample constraints, we were not able to perform HLA-phenotype analysis in our patients. Second, the immense promiscuity [49] and cross-reactivity [50] of TCRs does not preclude the presence of TCR responses to common autoantigens between these patients. Third, the functional identity of the dominant clones in these repertoires is largely unknown and may mostly be represented by the individual’s response to vaccinations and infections given that most of these clones are stably maintained at high levels irrespective of disease activity. In such scenarios, public TCR clones may exist in SLE but without achieving repertoire dominance. And finally, the phenomenon of epitope spreading [51–53] further diversifies the TCR response from its original epitope to newer epitopes over time. Public responses may be more likely in the preclinical stages of the disease when the immune response is directed toward a limited set of autoantigens [54].

Despite treatments, SLE patients exhibit an unpredictable disease course with no reliable biomarkers to predict flare. The clinical relevance of this study has been in its use of longitudinal samples from SLE patients with diverse manifestations and treatment backgrounds who flared mostly to moderate disease activity level. However, lack of knowledge of autoantigens or the autoreactive clones is a limiting factor and has hampered the use of such NGS data to track disease-specific clones. In this regard, when possible, concomitant TCR profiling from tissue biopsies where the immune response is dominated by fewer HECs could be performed to help pinpoint possible autoreactive clones.

## Conclusions

In summary, using NGS of TCRβ, we showed that SLE patients displayed about a twofold reduction in T cell repertoire diversity per fixed PB volume when compared to HC. The relative proportion of clonally expanded T cells was higher in SLE as compared to HC, although the difference did not reach statistical significance. Importantly, in most SLE patients, longitudinal analysis showed that diversity, the skewing of clonal distribution, or proportion of clonal expansion in the repertoire did not change significantly at disease flare relative to clinical quiescence. Overall, these results suggest that clonal expansions in the context of reduced repertoire may be a defining feature of SLE, however, increases in disease activity are not reflected with immunological changes at the total T cell repertoire level. Nevertheless, if disease-specific T cell clones can be identified, next-generation sequencing may provide a powerful and high-resolution approach to monitor pathogenic clones that may correlate better with disease activity in SLE.

## Additional files

Additional file 1: Table S1. Summary of TCRβ CDR3 sequencing results from all 51 samples. **Table S2.** Top 50 HC clones: CDR3 sequences, rank and frequency. Clones that show significant changes are marked in red. **Table S3.** Top 50 SLE clones: CDR3 sequences, rank and frequency. Clones that show significant changes are marked in red. Additional file 2: Figure S1. Cumulative contributions to the total repertoire by the top 500 clones are shown for HC (A) and SLE subjects (B). Dotted line represents the median value. **Figure S2.** Differential Vβ gene (A) and Jβ gene (B) usage between HC and SLE groups. Genes that are differentially expressed between the two groups are indicated by asterisk after the gene name (^*^, *P* <0.05; ^**^, *P* <0.01; *t* test, FDR = 10 %). Level of gene usage was calculated as a percentage of uniques. **Figure S3.** Longitudinal analysis of the proportion of repertoire occupied by the three clonal groups in SLE (top 50, >95^th^ percentile, <95^th^ percentile) during quiescence (A), pre-flare (B), and flare (C). The same data is shown in line graph form for the top 50 clones (D) and the top 5^th^ percentile of clones (E). **Figure S4.** Longitudinal analysis of the proportion of repertoire occupied by the three clonal groups in HC (top 50, >95^th^ percentile, <95^th^ percentile) during baseline (A) and at times 5 to 6 months later (B). The same data is shown in line graph form for the top 50 clones (C) and the top 5^th^ percentile of clones (D).

## Abbreviations

AZA: azathioprine
C3: complement component 3
C4: complement component 4
CDR3: complementarity-determining region 3
dsDNA: double-stranded DNA
FDR: false discovery rate
gDNA: genomic DNA
HC: healthy controls
HCQ: hydroxychloroquine
HEC: highly expanded clones
HLA: human leukocyte antigen
LFM: leflunomide
MMF: mycophenolate mofetil
NGS: next-generation sequencing
PB: peripheral blood
PGA: Physician Global Assessment
Pred: prednisone
RT-PCR: reverse transcriptase-polymerase chain reaction
SD: standard deviation
SLE: systemic lupus erythematosus
SELENA-SLEDAI: Safety of Estrogens in Lupus Erythematosus National Assessment - SLE disease activity index
SSCP: single-strand conformation polymorphisms
TCR: T cell receptor
WBC: white blood cell
