# Supplementary material for: Longitudinal analysis of peripheral blood T cell receptor diversity in patients with systemic lupus erythematosus by next-generation sequencing
Source: Arthritis Res Ther. 2015 May 23;17(1):132. doi: 10.1186/s13075-015-0655-9 (PMC4458014; doi:10.1186/s13075-015-0655-9)
Supplement: Additional file 2: Figure S1. — Cumulative contributions to the total repertoire by the top 500 clones are shown for HC (A) and SLE subjects (B). Dotted line represents the median value. Figure S2. Differential Vβ gene (A) and Jβ gene (B) usage between HC and SLE groups. Genes that are differentially expressed between the two groups are indicated by asterisk after the gene name (*, P <0.05; **, P <0.01; t test, FDR = 10 %). Level of gene usage was calculated as a percentage of uniques. Figure S3. Longitudinal analysis of the proportion of repertoire occupied by the three clonal groups in SLE (top 50, >95th percentile, <95th percentile) during quiescence (A), pre-flare (B), and flare (C). The same data is shown in line graph form for the top 50 clones (D) and the top 5th percentile of clones (E). Figure S4. Longitudinal analysis of the proportion of repertoire occupied by the three clonal groups in HC (top 50, >95th percentile, <95th percentile) during baseline (A) and at times 5 to 6 months later (B). The same data is shown in line graph form for the top 50 clones (C) and the top 5th percentile of clones (D). [file 13075_2015_655_MOESM2_ESM.docx]

**Supplementary Figure 1**

**A**

**B**

**Supplementary Figure 2**

**A**

**B**

**Supplementary Figure 3**

**A**

**D**

**B**

**E**

**C**

**Supplementary Figure 4**

**C**

**A**

**B**

**D**
